# Chemical Characterization of Different Products from the Tunisian *Opuntia ficus-indica* (L.) Mill.

**DOI:** 10.3390/foods11020155

**Published:** 2022-01-07

**Authors:** Ambrogina Albergamo, Angela Giorgia Potortí, Giuseppa Di Bella, Nawres Ben Amor, Giovanna Lo Vecchio, Vincenzo Nava, Rossana Rando, Hedi Ben Mansour, Vincenzo Lo Turco

**Affiliations:** 1 Department of Biomedical, Dental, Morphological and Functional Images Sciences (BIOMORF), University of Messina, Viale Annunziata, 98100 Messina, Italy; aalbergamo@unime.it (A.A.); agpotorti@unime.it (A.G.P.); giovanna.lovecchio@unime.it (G.L.V.); vnava@unime.it (V.N.); rrando@unime.it (R.R.); vloturco@unime.it (V.L.T.); 2 Research Unit of Analysis and Process Applied to Environmental-APAE UR17ES32, Higher Institute of Applied Sciences and Technology of Mahdia, University of Monastir, Monastir 5000, Tunisia; nawres_benamor@yahoo.com (N.B.A.); hdbenmansour@gmail.com (H.B.M.); 3 National Institute of Applied Sciences and Technology (INSAT), University of Carthage, Carthage 1054, Tunisia

**Keywords:** Tunisian *O. ficus-indica*, nopal, fruit pulp, peel, seeds, by-products, inorganic elements, polyphenols, fatty acids, sugars

## Abstract

Various dried (by-)products from the Tunisian *O. ficus-indica* were elucidated for their proximate composition, fatty acid (FA) composition, inorganic elements, sugars, and polyphenols. Nopal and prickly pear peel and seeds were abundant in fiber (respectively, 28.39, 12.54, and 16.28%). Seeds had also high protein (17.34%) and may be source of an edible oil, due to lipids (9.65%) poor in saturated FAs (14.12%) and rich in linoleic acid (61.11%). Nopal and peel showed the highest levels of Mg (493.57 and 345.19 mg/100 g), K (6949.57 and 1820.83 mg/100 g), Mn (59.73 and 46.86 mg/Kg) and Fe (23.15 and 15.23 mg/Kg), while the fruit pulp predominantly constituted of sugars, glucose and arabinose being predominant (42.57 and 13.56 g/100 g). Total polyphenols widely varied among the Opuntia products (108.36–4785.36 mg GAE/100 g), being mainly represented by hydroxycinnamic and hydroxybenzoic acids, and flavonoids as well. In particular, peel may be revalorized for these valuable bioactives, including 4-hydroxybenzoic acid (484.95 mg/100 g), cinnamic acid (318.95 mg/100 g), rutin (818.94 mg/100 g), quercetin (605.28 mg/100 g), and several isorhamnetin and kaempferol glycosides. Overall, the Tunisian prickly pear cactus could encourage a sustainable production, an effective waste management, and may provide several benefits for human health, in accordance with the model of the Mediterranean diet.

## 1. Introduction

With the global population growth, health experts, policy makers, and not least consumers, are increasingly aware that food resources must be optimized as much as possible by ensuring their sustainable use both at the industrial and household level, while taking advantage of their potential health benefits. Hence, synergies between policies and interventions have been recently created to reduce the environmental impact of agri-food chains and promote consumer wellness and health by means of suitable dietary patterns [1].

Recognized as an intangible cultural heritage with peculiar lifestyle and eating habits, the Mediterranean diet could address both health and environmental concerns, as it is essentially based on the greater use of local and in-season plant-based foods and the lower consumption of animal products. In fact, on the one hand, it provides precious fiber and minerals, a high monounsaturated-saturated fat ratio, and a balanced n-6/n-3 ratio as well [2,3,4,5,6,7,8,9,10,11,12] which, according to the increasing scientific evidence, would be responsible for a lower risk of cardiovascular mortality and coronary diseases, diabetes, metabolic syndrome, and certain neurodegenerative disorders and cancers [13]. On the other hand, the Mediterranean diet is marked by a reduced environmental impact—in terms of production of waste and emissions—and preserves the biodiversity of the Mediterranean basin itself [14,15].

*Opuntia ficus-indica* (L.) Mill., commonly known as the prickly pear cactus, is native to Mexico and has subsequently propagated in Latin Americas, South Africa, and the Mediterranean area, where many ecotypes, cultivars, and clones of the species have been established between the XVII and XIX centuries, especially in Tunisia. In this country, 600,000 ha of *O. ficus-indica* have been planted in the last 50 years, and the cactus species has proved to be an important fodder crop principally in the central and southern regions, during periods of drought and seasons of low feed availability [16,17].

The prickly pear cactus has well adapted to the Mediterranean diet pattern by establishing itself initially as wild plant and, later, as a crop for market-oriented agriculture and sustainable agricultural systems as well [18,19]. Under the marketing perspective, the prickly pear cactus is recognized for the seasonal and profitable production of fleshy fruits—called prickly pears—characterized by acceptable organoleptic properties, and excellent nutritional and health properties owed to the presence of fiber, minerals, vitamins, and antioxidant compounds (i.e., phenols, flavonoids, betaxanthin, and betacyanin). Overall, fruits and derived products, such as juice, jam, and syrup, are increasingly appreciated, thus, contributing to enrich the already varied Mediterranean diet [20,21].

In terms of sustainability, the cultivation of prickly pear cactus may counteract the climate change and desertification, promote water conservation, and protect local fauna, by serving as a lifesaving crop [22]. Additionally, many efforts have been devoted to the reclamation of waste from agricultural and processing practices, such as cladodes, fruit peels, and seeds, which—besides having been already used in folk medicine for their nutritional properties (i.e., antioxidant effects) and beneficial activities (i.e., anti-inflammatory hypoglycemic, neuroprotective effects etc.) [23]—have a considerable impact on the supply chain of *Opuntia* [24,25]. In fact, around 6–8 tons/hectares of cladodes—or simply nopal—are produced annually following the pruning period, which are not easy to be disposed of, and represent one of the main costs for farmers [26]; while peels and seeds, derive from the industrial processing occurring mostly during the fruiting period, constitute 45–55% and 2–10%, respectively of the total weight of the fruit, and end up either landfilled or employed as animal feed [27]. The possibility of turning such wastes into high added-value by-products has implied not only a reduction in the crop management costs, but also income diversification for farmers. Indeed, nopal has already been reused in a variety of products, such as fortified food, cosmetics, biopolymers, and flocculating agents for treating water and wastewater [28,29,30]. Peels have been valorized by the production of fortified foods, nutraceuticals, pharmaceuticals, natural colorants, and zinc oxide nanoparticles [27,31,32,33], while seeds have been reclaimed for the production of an edible oil with a high unsaturation degree [34], as well as a protein flour used to enrich bread and rice-based extrudates [35,36,37]. In every case, the prior knowledge of the plethora of phytomolecules constituting such by-products continues to be mandatory for explaining their technological, functional, and healthy attributes and, consequently, for researching and valorizing their potential reuses.

Within this context, aim of this study was to chemically characterize the nopal and the fruit—pulp, peel, and seeds—from the Tunisian *O. ficus-indica*, in an attempt to make the most of its potential in the context of the Mediterranean diet model.

## 2. Material and Methods

### 2.1. Sample Collection

Spiny cladodes and fruits from *Opuntia ficus-indica* (L.) Mill., were hand-picked from naturalized plantations located in the coastal area of Mahdia governorate (North-Eastern Tunisia) during August 2020. The taxonomic identification of the species was performed by the botanists of the University of Monastir (Monastir, Tunisia), where a voucher specimen was also deposited.

**Figure 1:**
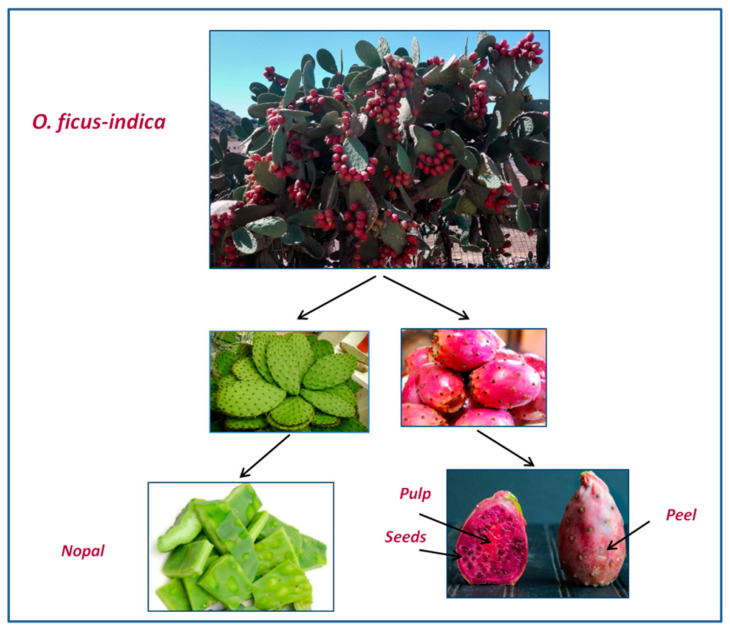
Spiny *O. ficus-indica* from Mahdia governorate (Tunisia) and collected plant parts employed for the study.

Specifically, three samples of fresh nopal, where each sample consisted of *n* = 10 adult cladodes with an approximate length of ~50 cm, and three samples of fresh prickly pears, each comprising *n* = 15 ripe fruits with red pulp, were collected, washed and dethorned (Figure 1). Every fruit sample was further peeled, and the pulp was manually separated from seeds to obtain respectively three samples of pulp, three samples of peels, and three samples of fruit pulp (Figure 1).

Hence, fresh samples from nopal, pulp, peel, and seeds were separately cut in small slices (seeds excluded) and freeze-dried by an Alpha-LD Plus freeze dryer (Martin Christ, Osterode, Germany), for 72 h at −55 °C using a chamber pressure of 0.110 mbar. Then, every dried sample was weighed, grinded (Retsch Grindomix GM200, Retsch GmbH, Haan, Germany) and passed through a 20 mm sieve to obtain fine powders. The average moisture content removed from nopal, pulp, peel, and seed samples was respectively 85, 75, 73, and 5%. Samples were stored at −18 °C until analysis.

### 2.2. Chemicals and Reagents

For crude protein, the Kjeldahl catalyst was supplied by Carlo Erba (Milan, Italy). Solvents, including *n*-hexane and *n*-heptane (reagent grade), and acetonitrile and methanol (HPLC grade), were from J.T. Baker (Phillipsburg, NJ, USA). Hydrogen peroxide (30% *v*/*v*) and nitric acid (65% *v*/*v*) (trace metal analysis grade) and ultrapure water (resistivity of 10 mΩ cm) were from Mallinckrodt Baker (Milan, Italy). Reference standards of fatty acids methyl esters (FAMEs, C4–C24) were purchased from Supelco (Bellefonte, PA, USA). Stock solutions of various inorganic elements (1000 mg/L in 2% HNO_3_, each) were supplied by Fluka (Milan, Italy), while reference standards of individual sugars and polyphenols were obtained from Sigma-Aldrich (St. Louis, MO, USA) and Extrasynthese (Genay, France).

### 2.3. Proximate Composition

The determination of proximate composition of the several *Opuntia* samples was carried out according to the AOAC official protocols of analysis [38]. For every sample, dietary fiber was determined following the AOAC Official Method 991.43 and the AACC Method 32-07.01, by employing the Megazyme assay kit (International Ireland Ltd., Wicklow, Ireland). Briefly, two sample aliquots (1.0 g each) were treated in parallel by α-amylase at 80 °C and then digested with protease and amyloglucosidase at 60 °C. The solutions were cooled at ~40 °C and treated with ethanol to precipitate fiber. The residues were filtered, washed with organic solvents, dried and the mean weight was calculated. At this stage, a residue was incubated at 500 °C until constant weight (~12 h) for determining ash, whereas the other residue was analyzed for crude protein by the AOAC Official Method 976.05. In this case, the residue was digested with sulfuric acid, copper (II) selenite dihydrate, and potassium sulfate by the SpeedDigester K-439 (Büchi, Switzerland) and then analyzed by the KjelMaster System K-375 equipped with a scrubber of gases and vapors (Büchi, Switzerland). The resulting solution was treated with sodium hydroxide, to develop ammonia. The nitrogen amount was evaluated by titration with hydrogen chloride, and the calculation of the protein (%) occurred by multiplying the nitrogen (%) by a conversion factor of 6.5. Hence, dietary fiber was equal to the mean weight of the dried residue less the weight of protein and ash. For total fat, the AOAC method 920.39 was exploited. Briefly, a sample aliquot (15.0 g) was extracted by a Soxhlet apparatus with *n*-heptane for 6 h. Then, the extract was dried by a rotating evaporator (Heidolph Instruments GmbH & Co., Schwabach, Germany), and the extraction yield was gravimetrically determined. Moisture was evaluated according to the AOAC method 925.09 by oven drying the sample at 110 °C for 4 h and subsequently registering the sample weight loss, while total carbohydrates were calculated by difference. For every component, every *Opuntia* sample was analyzed in triplicate.

### 2.4. Fatty acid (FA) Profile

Samples from nopal, prickly pear pulp, peel, and seeds were investigated for the FA composition according to that which was already reported by Di Bella et al. [24]. Every lipid extract obtained by the Soxhlet apparatus was transmethylated by a “warm” transesterification and injected into a gas chromatograph (GC) equipped with a split/splitless injector and coupled to a flame ionization detector (FID) (Dani Master GC1000, Dani Instrument, Milan, Italy). For the chromatographic separation, a Supelco SLB-IL100 capillary column (60 m × 0.25 mm ID, 0.20 μm film thickness, Supelco, Sigma Aldrich, USA) was used, with an oven temperature program from 130 to 210 °C (10 min holding) at 2 °C/min; injector and detector temperatures at 220 and 240 °C respectively; helium (He) at a linear velocity of 30 cm/s (constant) and an initial head pressure of 99.5 kPa, and sampling frequency: 12.5 Hz. Other experimental conditions were hydrogen gas (H_2_) 40 mL/min, makeup gas (He) 30 mL/min and air 300 mL/min. The injection volume was 1 μL, with a split ratio of 1:100. Data analysis was performed by the instrumental software (Clarity Chromatography, v.4.0.2). FAMEs of nutritional interest were identified by direct comparison with the retention times of reference compounds, while the quantitative analysis occurred by calculating the individual FAME percentage with respect to the total chromatogram area. Every sample was analyzed in triplicate along with analytical blanks.

As already reported in Dimić et al. [39], the dietary quality of fat from *Opuntia* (by-)products was determined by three indices derived from the relative from FA profiles, such as the ratio between hypocholesterolemic and hypercholesterolemic FAs (H/H):

(1) $$\frac{H}{H}=\frac{C18:1+C18:2+C18:3}{C14:0+C16:0}$$

the atherogenicity index (AI):

(2) $$\mathrm{AI}=\frac{C14+4\left(C16:0\right)}{\sum \mathrm{MUFA}+\sum \omega 3+\sum \omega 6}$$

and thrombogenicity index (TI):

(3) $$\mathrm{TI}=\frac{C14:0+C16:0+C18:0}{0.5\left(\sum \mathrm{MUFA}\right)+3\left(\sum \omega 3\right)+0.5\left(\sum \omega 6\right)+\left(\sum \omega 3/\sum \omega 6\right)}$$

where, C14:0 is myristic acid, C16:0 is palmitic acid, C18:0 is stearic acid, C18:1 is oleic acid, C18:2 is linoleic acid, and C18:3 is α-linolenic acid. ∑MUFA is the sum of monounsaturated FAs, Σω-3 is the sum of the polyunsaturated ω-3 FAs, and Σω-6 is the sum of the polyunsaturated ω-6 FAs.

Additionally, another important determinant of health, namely the ratio of ω-6/ω-3 essential FAs, was evaluated [40].

### 2.5. Inorganic Elements

For every Opuntia (by-)product, a sample aliquot (0.5 g) was digested with 2 mL of H_2_O_2_ and 7 mL of HNO_3_ with a closed-vessel microwave digestion system (Ethos 1, Milestone, Bergamo, Italy). A temperature program, consisting of 0–200 °C in 10 min (step 1), and 200 °C held for 15 min (step 2), with a constant microwave power of 1500W, was adopted. The screening of minerals (i.e., Na, Mg and K), trace essential elements (i.e, Mn, Fe, Cu, and Zn), and potentially toxic elements (i.e., Ni, Cr, As, Cd and Pb) was performed by a quadrupole ICP-MS (iCAP Q, Thermo Scientific, Waltham, MA, USA), according to our optimized method already used for a number of plant matrices [41,42,43,44,45,46]. The instrumental conditions of analysis were incident radio frequency (rf) power 1500 W; plasma gas flow rate (Ar) 15 L/min; auxiliary gas flow rate (Ar) 0.9 L/min, and carrier gas flow rate (Ar) 1.10 L/min. The ICP-MS was operated with helium as collision cell gas at a flow rate of 4 mL/min and was equipped with a spray chamber (2 °C). The volume of injection and the flow rate of sample introduction were 200 μL and 1 mL/min, respectively. Spectra acquisition occurred in full scan mode (dwell time 0.5 or 0.1 s/point, based on the analyte). Data analysis was performed by the instrumental software (Qtegra™ Intelligent Scientific Data Solution). Triplicate measurements along with analytical blanks were carried out for every sample. For quantification, external six-point calibration curves were built up for each analyte (*r*^2^ ranging from 0.9999 [Mg, K and Cr] to 0.9991 [Cu]). The performance of the ICP-MS procedure was evaluated with the help of commercial standards in terms of linearity, limit of detection (LOD), and quantification (LOQ), intra- and interassay variability and accuracy. (See Supplementary Material for further details).

### 2.6. Sugars

Every Opuntia (by-)product was screened for sugars following the ISTISAN method 96/34 described by Baldini et al. [47]. Briefly, a sample aliquot (10.0 g) was mixed with 100 mL of acetonitrile/water (60:40, *v*/*v*), and stirred, first, for 30 min by an ultrasound bath and then for 3 h by a magnetic stirrer. The obtained supernatant was filtered, purified by a C18 Sep-Pak cartridge (Waters, Milford, MA, USA), and, analyzed by high-performance liquid chromatography coupled to a refractive index detector (HPLC-RI), employing a Shimadzu LC10A System (Shimadzu, Kyoto, Japan) coupled to a Shimadzu RID-6A RI detector (Shimadzu, Kyoto, Japan). The chromatographic separation occurred with a reverse phase propyl-amine-based column (Supelcosil LC-NH2, 25 cm × 4.6 mm, 5 μm ID, Supelco, Bellefonte, PA, USA) and a mobile phase consisting of acetonitrile/water (50:50), with a flow rate of 1 mL/min. The injection volume and the column oven temperature were set at 10 µL and 25 °C, respectively. All samples were analyzed in triplicate along with analytical blanks. For quantification, six-point calibration curves were constructed for every investigated sugar (*r*^2^ ranging from 0.993 [arabinose] to 0.998 [glucose]).

### 2.7. Total and Single Polyphenols

An aliquot (3.0 g) of every *Opuntia* sample was suspended in 10 mL of aqueous methanol (80%) and stirred overnight in ice. Then, the mixture was centrifuged at 10,000× *g* for 15 min at 4 °C and the resulting supernatant was collected for analysis. Total phenol content (TPC) was assessed by the method proposed by Dewanto et al. [48] with slight modifications. Briefly, 10 µL of supernatant were added with 240 µL of distilled H_2_O and 250 µL of Folin-Ciocalteau reagent. The solution was stirred, and further mixed with 2.5 mL of 7% NaHCO_3_, to obtain a final volume of 3 mL. The resulting solution was kept in the dark for 90 min at room temperature, and subsequently analyzed with the UV–VIS spectrophotometer (UV-2401 PC, Shimadzu, Milan, Italy) at an absorbance wavelength of 760 nm. A six-point calibration curve was constructed using appropriate dilutions of a standard solution of gallic acid (*r*^2^ = 0.9907). As a result, the total polyphenol content was calculated as mg of gallic acid equivalent per 100 g of dried sample (mg GAE/100 g). All determinations were carried out in triplicates along with blank solutions.

For single polyphenols, sample aliquots (3.0 g) of nopal, fruit pulp and peel, as well as an aliquot (3.0 g) of defatted powder from seeds, were mixed with 10 mL of aqueous methanol solution (80%) and stirred overnight in an ice bath. The resulting mixtures were centrifuged at 10,000× *g* for 15 min (+4 °C) and the obtained supernatants were filtered through 0.45 μm and 0.20 μm polytetrafluoroethylene (PTFE) filters. The analysis occurred by HPLC coupled to diode-array detection and mass spectrometry (HPLC-DAD-MS), exploiting an LC apparatus (Prominence UFLC XR system, Shimadzu, Kyoto, Japan), equipped with a controller (CBM-20A), binary pumps (LC-20AD-XR), degasser (DGU-20A3R), PDA detector (SPD-M20), column oven (CTO-20AC), and autosampler (SIL-20A XR). The method of analysis was already reported by Albergamo et al. [49] and involved the use of an Ascentis Express C18 (250 × 4.6 mm inner diameter × 2.7 μm particle diameter; Supelco) and of mobile phases, such as water/formic acid (99.9:0.1, *v*/*v*) (solvent A) and acetonitrile/formic acid (99.9:0.1, *v*/*v*) (solvent B). The gradient of elution was 0 min, 5% B, 5 min, 5% B, 15 min, 30% B, 40 min, 60% B, 45 min, including steps such as column washing and re-equilibration. The mobile phase flow rate was equal to 1.0 mL/min, whereas the injection volume and the oven temperature were 5.0 μL and 30 °C, respectively. The PDA spectra were acquired in the range 190–400 nm, and the chromatograms were extracted with wavelengths between 280 and 370 nm (time constant 0.60 s; sample frequency 1.5625 Hz). An electrospray ionization (ESI) source interfaced the LC system to a triple quadrupole mass spectrometer (MS) (LCMS-8040, Shimadzu, Kyoto, Japan). A splitting device (VICI AG International, Schenkon, Switzerland) assured that only one-third of the total flow was directed from the LC system to the ESI interface. The MS acquisition was performed with ESI interface in negative mode, and MS operating in full-scan (*m*/*z* 100–800) and selected ion monitoring (SIM) modes, with the following conditions: ESI temperature, 350 °C; heat block, 300 °C and desolvation line (DL) temperature, 300 °C; scan speed 715 amu/sec; nebulizing gas (N_2_) flow 1.5 L/min; drying gas (N_2_) flow 10 L/min. Every *Opuntia* sample was analyzed in triplicate along with analytical blanks. Data analysis was performed by the instrumental software (LabSolution, v. 5.53).

The investigated compounds belonged to the classes of hydroxybenzoic acids, hydroxycinnamic acids, and flavonoids (flavones and flavonols). They were selected depending on standard availability and their characteristic occurrence in the different products from *O. ficus-indica* [50,51,52]. For quantification purposes, an external calibration procedure was conducted by means of six-point calibration curves (*r*^2^ ranging from 0.9947 [kaempferol 3-O-glucoside] to 0.9998 [luteolin]). The LC-MS method was successfully validated in terms of linearity, LOD, LOQ, accuracy, intra- and interassay variability (See Supplementary Material for further details).

### 2.8. Statistical Analysis

Experimental data were expressed as mean ± standard deviation of three replicate measurements per sample. Statistical analyses were performed using the SPSS package (SPSS 21.0, Chicago, IL, USA).

Statistically significant differences in proximate composition, FA composition, inorganic elements, sugars, and total and single polyphenols over the different parts of the Tunisian *O. ficus-indica* were verified by one-way analysis of variance (ANOVA), followed by a post hoc Tukey’s honestly significant difference (HSD). For every investigated variable, statistical significance was accepted at *p* < 0.05.

## 3. Results and Discussion

### 3.1. Proximate Composition

The different (by-)products from *O. ficus-indica* showed peculiar proximate compositions (Table 1). Overall, nopal and peel were characterized by similar and quite low protein content (1.36 and 1.22%, respectively; *p* > 0.05), with respect to the fruit pulp and seeds, which showed, respectively, the lowest and the highest crude protein (0.78 and 17.34%, respectively; *p* < 0.05). Lipids were most abundant in seeds and peel (9.65 and 5.04%; *p* < 0.05), and lower and non-significantly different in nopal and fruit pulp (1.15 and 1.12%, *p* > 0.05). Nopal exhibited the highest amount of dietary fiber (28.39%, *p* < 0.05), followed by seeds and peel (16.28 and 12.54%, respectively; *p* > 0.05); whereas the fruit pulp was marked by the lowest levels of such component (4.06%, *p* < 0.05). Ash ranged from 18.58% in nopal to 0.28% in pulp (*p* < 0.05). Carbohydrates were significantly different in all samples (*p* < 0.05), comprising between 74.34% (in pulp) and 38.79% (in nopal) (Table 1).

Based on the obtained results, the Tunisian nopal, as well as prickly pear peel and seeds, may be labeled as a “natural source of fiber”, because the results showed they were naturally characterized by more than 3% of dietary fiber, according to the Regulation (EC) 1924/2006 [53]. Additionally, prickly pear seeds may serve as a high protein source. In this respect, when compared with other seeds, the protein content of prickly pear seeds is similar to that of chia seeds (18.81%), flaxseed (20.30%), rapeseed (19.00 g/100 g), and sesame (15.07%) [54].

To the best knowledge of the authors, the proximate composition of the different (by-)products of the Tunisian *O. ficus-indica* has not been yet elucidated elsewhere. Only one previous work [55] focused on the chemical characterization of Tunisian dried nopal, reporting, an improbable proximate composition (protein: 8.74%; fat: 3.95%; ash: 25.65%; dietary fiber: 51.24%, and carbohydrates: 60.36%). Overall, the Tunisian nopal was marked by notably different level of protein, carbohydrates, and dietary fiber and similar content of lipid and ash when compared to the Mexican nopal, which literature predominantly referred to. In fact, dried nopal from different cultivars (i.e., Blanco, Manso, Amarillo, and Cristalino) showed higher protein (6.7–19%), carbohydrates (55.1–66.5%), and dietary fiber (6.2–15%), while lipids and ash were, respectively, equal to 0.1–1.5% and 14.8–18.8% [56]. Interestingly, dried nopal from different Sicilian cultivars had protein (1.48–2.18%), ash (12.81–15.23%), and lipids (0.76–2.46%) similar to the Tunisian counterpart. However, it was characterized by higher levels of dietary fiber (41.10–46.72%) [24].

Contrary to nopal, literature reports a greater consistency with results obtained from the pulp of the Tunisian prickly pear. In fact, the dried pulp of Algerian, Canarian, and Egyptian prickly pears was overall characterized by protein equal to 0.87–1.62%, lipids 0.48–0.75%, ash 0.4–2.6% and dietary fiber 4.65–5.37% [57,58,59].

Dried peels from Algerian and Egyptian prickly pears showed protein and ash contents comparable to that from Tunisian prickly pear peels (protein: 0.90–1.53% and ash: 3.05–3.4%) [57,59,60]. However, no consistency was observed for the other proximate components, which were found at much lower levels (lipids: 0.32–1.69%, fiber: 0.96–8.53% [57,60,61].

*Opuntia* seeds coming from different countries (Algeria, Ethiopia, and Turkey), and dried in an oven or under atmospheric conditions, reported a fiber content comparable to that from Tunisian seeds (12.47–18.23%). Nevertheless, protein (4.48–10.00%) and lipids (3.66–5.00%) were lower than the counterparts from this study, thus confirming that Tunisian *Opuntia* seeds are valuable sources of protein and fat [57,62,63].

### 3.2. Fatty Acid (FA) Profile

The FA composition of various (by-)products from the Tunisian *Opuntia* is reported in Table 2.

Overall, nopal and peels were characterized by the highest amounts of polyunsaturated FAs (PUFAs, 56.69 and 60.21%), followed by saturated FAs (SFAs, 23.79 and 23.42%) and MUFAs (19.41 and 16.21%). On the other hand, pulp and seeds showed a greater unsaturation degree, as PUFAs were the most abundant (52.93 and 61.71%), followed by MUFAs (25.05 and 22.42%) and SFAs (21.94 and 14.12%) (Table 2).

Although every matrix revealed a peculiar FA profile, in general, palmitic acid (C16:0) was the most abundant SFA (from 10.35% in seeds to 21.14% in nopal), oleic acid (C18:1 n-9, from 13.56% in peels to 23.26% in fruit pulp) exhibited the 83–93% of total MUFAs, while linoleic acid (C18:2 n-6) was the predominant PUFA (from 36.44% in nopal to 61.11% in seeds), representing up to 99% of total PUFAs in seeds (Table 2). In particular, prickly pear seeds reported the FA composition typical of an edible seed oil with potential health benefits, due to the abundant presence of PUFAs, especially the essential linoleic acid, and the lowest content of SFAs (14.12%) [62,64,65]. Indeed, similar FA profiles have been already reported for the grape seed oil (C16:0, 9.91%; C18:0, 2.88%; C18:1 n-9, 26.51%; C18:2 n-6, 53.84%,) [66], the paprika seed oil (C16:0, 13.8%; C18:0, 3.7%; C18:1 n-9, 14.6%; C18:2 n-6, 67.8%) [65], and the niger seed oil (C16:0, 12.0%; C18:0, 3.0%; C18:1 n-9, 13.5%; C18:2 n-6, 65.4%) [65].

The dietary quality of fat from different *Opuntia* (by-)products was assessed by the H/H ratio, AI, and TI indices (Table 2), namely indicators of the stimulation of cholesterol metabolism and platelet aggregation responsible for cardiovascular syndromes [67].

H/H values ranged from 3.48 (nopal) to 7.95 (seeds). In fatty matrices, such as *Opuntia* seeds, a higher level of this index—directly proportional to the PUFAs content—would be desirable, as it expresses the effect of FAs on cholesterol metabolism. In this respect, comparable H/H values were found in the sesame oil (7.72), while linseed (13.24), grapeseed oils (11.07–12.28), hemp (14.97), and flax (17.05) seed oils showed higher H/H ratios [40,61,68].

*Opuntia* (by-)products showed low AI and TI indices—being directly proportional to SFAs—and, as a result, had a good potential for protection against coronary diseases. Specifically, AI was comprised of between 0.49 (seeds) and 1.11 (nopal), while TI was in the range 0.27 (nopal) and 0.43 (pulp). Overall, a comparable AI was found in pomegranate seed oil (0.42) [61]; while linseed (0.00) and grapeseed (0.081–0.090), hemp (0.07) and flax (0.06) oils were marked by even lower AIs [40,61,68]. Hemp (0.10), flax (0.05) and linseed (0.04) oils showed even lower Tis, while sesame (0.26), grapeseed oils (0.24–0.26), and pomegranate oil (0.75) had comparable and higher Tis, respectively, than the investigated products [40,61,68].

Considering the ω-6/ω-3 ratio, the Tunisian nopal showed the most balanced ratio (1.89), followed by peels (4.27). As expected, seeds had a very imbalanced ratio (211.83), due to a very high content of ω-6 FAs and very small amounts of ω-3 FAs. Overall, literature reported oils from hemp, flax seeds, and linseed with optimal ω-6/ω-3 ratios (respectively, 3.79, 0.28, 0.22) *Opuntia* seeds [61,68]. However, sesame oil showed an unbalanced ω-6/ω-3 ratio, being equal to 50 [69].

To the best knowledge of the authors, the FA composition of the different (by-)products of the Tunisian *O. ficus-indica* has not been yet investigated elsewhere.

Literature reported a general disagreement among the few investigations on Mexican, Spanish, and Sicilian nopal and the results from this study. With respect to the nopal from this study, Mexican dried nopal was marked by higher palmitic (20.5–36.4%) and linolenic (21.58–26.4%) acids, as well as lower oleic (9.48–11.03%) and linoleic (25.05–28.61%) acids [70]. On the other hand, Spanish dried nopal from six cultivars showed a highly variable FA composition depending on the cultivar investigated (palmitic acid: 30.1–50.0%; oleic acid: 9.22–22.3%; linoleic acid: 12.8–27.9%; linolenic acid: 5.31–11.5%) [71]. Recently, Di Bella and colleagues [24] pointed out that dried Sicilian nopal had much higher levels of palmitic acid (32.07–36.40%), but lower amounts of oleic (13.00–15.47%), linoleic (21.43–24.27%), and linolenic (11.02–15.61%) acids.

With respect to the fruit pulp, a greater consistency between literature and findings from this study was observed. In this respect, the dried *Opuntia* fruit pulp from six Spanish cultivars reported the following ranges: palmitic acid 14.8–29.0%, linoleic acid 20.2–53.39%, and linolenic acid 10.8–20.8% [71]. The dried pulp from the Egyptian *Opuntia* showed the same predominant FAs to be within the ranges discussed above [59].

Considerable attention has been given to the investigation of the FA profile of prickly pear peels and seeds.

In particular, dried peels from Egyptian, Spanish, and commercial *Opuntia* showed highly changing levels of palmitic (15.76–32.1%) and oleic (6.83–24.1%) acids, as well as linoleic (28.96–52.00%) and linolenic (0.43–21.9%) acids [59,60,71,72].

However, similarly to fruit pulp, *Opuntia* seeds with different origins showed a quite homogenous FA composition. Indeed, Turkish, Tunisian, and Algerian seeds dried under atmospheric conditions had predominant FAs such as palmitic acid comprised of between 9.23 and 13.4%, oleic acid from 13.0 to 25.52%, and linoleic acid between 49.3 and 63.1% [62,64,73,74]. In particular, such PUFA reached up to 70.3% in seeds from the governorate of Sfax (Tunisia) [73]. Overall, the investigated seeds from the Mahdia governorate showed a FA composition comparable to that already described in literature.

### 3.3. Element Profile

The element profile of the different parts of the Tunisian *O. ficus-indica* is reported in Table 3. Among the investigated (by-)products, nopal was richest in minerals and essential trace elements, followed by peel, seeds, and pulp. All the matrices resulted a precious source of Mg and K, which significantly varied between 152.84–493.57 mg/100 g, and 187.05–6949.57 mg/100 g, respectively (*p* < 0.05). Among essential trace elements, Mn (0.78–59.73 mg/Kg, *p* < 0.05) and Fe (2.36–23.15 mg/Kg, *p* < 0.05) stood out in every investigated part. A reversed trend was observed for potentially toxic trace elements, as the fruit pulp resulted the most “contaminated” product, followed by nopal, peel, and seeds. In this respect, Ni and Cr were the most abundant elements, ranging from 33.28 to 47.14 µg/Kg (*p* < 0.05) and from 11.92–41.50 µg/Kg (*p* < 0.05). However, heavy metals such as Cd and Pb were at low and safe levels (11.28–25.62 µg/Kg, *p* > 0.05) and As was even <LOD (<0.015 µg/Kg) in every sample. Particularly, the various parts of the Tunisian prickly pear were within the limits established by the European Reg. 1881/2006 for Cd (0.050 mg/Kg, on a fresh weight basis) and Pb (0.10 mg/Kg, on a fresh weight basis) for what concerns fruits [75].

Although the element pattern of a vegetable matrix may greatly vary even within a given species, due to factors such as geographical origin, growth scenario, genetic variability, as well as maturity stage, the peels from the Tunisian *Opuntia* showed mineral levels similar to those from potato, carrot, and radish peels. Concerning essential trace elements, the investigated peels may be compared to the above-listed peels for the Mn content, but not for Fe and Zn, which resulted at much lower amounts [76]. On the other hand, the prickly pear seeds showed a mineral profile similar to that of watermelon, pumpkin, and rapeseed seeds, although essential trace elements, such as Fe, Zn, and Mn, were at much lower levels [77,78,79,80]. Overall, these considerations highlight the potential role of such by-products as useful ingredients for food fortification, and development of nutraceuticals as well.

In literature, minerals and trace elements of nopal were mainly investigated with respect to the Mexican *O. ficus-indica*, resulting, in general, in highly variable and not comparable contents with the Tunisian nopal. Indeed, the dried nopal from the Mexican cv. Redonda was marked by Mg varying from 880 to 1120 mg/100 g, Na 30–550 mg/100 g, and Mn 290–300 mg/Kg depending on the maturity stage [81]. Similarly, dried nopal from the other two Mexican cultivars (i.e., Milpa Alta and Atlixico) was characterized by higher Mg levels (1016.68–724.93 mg/100 g) than the Tunisian counterpart. However, elements such as K (5469.16–5648.15 mg/100 g), Na (83.12–112.45 mg/100 g) and Fe (31.6–34.3 mg/Kg) were found at similar levels to those described in this work [82].

The pulp of the Tunisian prickly pear was more abundant in minerals and essential trace elements than Kenian, Algerian, and Canarian dried fruits, marked by Mg and K in the range 1.05–76.10 mg/100 g and 11.14–559 mg/100 g, and Mn, Fe, and Zn varying between 3.0–69.9 mg/Kg, 2.0–33.5 mg/Kg, and 15.5–16.3 mg/Kg [57,58,83,84].

Similarly to pulp, the peels and seeds of the Tunisian fruits revealed higher concentrations of minerals than peels and seed derived from Turkish, Egyptian, Arabian, Ethiopian, and Algerian dried prickly pears (Mg varying between 1.47–322 mg/100 g in peels and 8.07–208 mg/100 g in seeds; K changing between 53.27–275 mg/100 g in peels and 53.27–275 mg/100 g in seeds [57,59,60,61,62,85]. However, the prickly pear peels and seeds previously investigated were characterized by higher trace essential elements than the Tunisian ones from this study (Mn: 180–729 mg/Kg in peels and 1.9–8.3 mg/Kg in seeds, Fe: 83.l-255.8 mg/Kg in peels and 11.7–121.0 in seeds [62,63,85].

### 3.4. Sugars

The sugar composition of the total carbohydrates from various Tunisian *Opuntia* products is reported in Table 4. Overall, every sample was characterized by a peculiar sugar profile, the prickly pear pulp being characterized by the highest concentration of almost all the monosaccharides, followed by peel, seeds, and nopal. Among investigated sugars, glucose was the most abundant sugar (8.75–42.57 g/100 g, *p* < 0.05), followed by xylose (7.79–22.29 g/100 g, *p* < 0.05) and arabinose (2.27–13.78 g/100 g, *p* < 0.05) (Table 3).

In dehydrated nopal, in particular, galactose (9.76 g/100 g) and arabinose (13.78 g/100 g) typically serve as monomers of a carbohydrate polymer, known as “opuntiamannan”, which show antihyperlipidemic and antihyperglycemic effects, by making dietary fat and sugar not absorbable through the intestinal tract [86].

The sugar composition of total carbohydrates from dried (by-)products from *O. ficus-indica* was poorly and fragmentarily elucidated only with regard to nopal and seeds. In fact, fruits and peels were mostly elucidated for the sugar composition of the pectin fraction [87,88].

Two previous works analyzed the monomeric sugars of dried cladode from the Calabrian and Sicilian *O. ficus-indica*, reporting a marked abundance in glucose (9.30–15.31 g/100 g), comparable levels of mannose (0.67–3.49 g/100 g), and lower levels of galactose (3.36–4.83 g/100 g), xylose (1.86–2.01 g/100 g), and arabinose (1.47–3.96 g/100 g) [24,89].

The dried seeds from Moroccan *Opuntia* showed higher levels of glucose and xylose than the seeds from this study (40.6 and 44.8%). However, galactose, arabinose and mannose were reported at similar levels (1.0, 3.1, and 1.0%, respectively) [90].

### 3.5. Polyphenols

According to the data provided by the Folin–Ciocalteu assay in Table 5, the highest total polyphenol content was detected in the peels of the prickly pears (4785.36 mg GAE/100 g). Although significantly different (*p* < 0.05), high total phenols were revealed also in cladodes (4235.27 mg GAE/100 g) and in the fruit pup (2381.68 mg GAE/100 g), while the seeds showed the lowest levels of these phytomolecules (28.36 mg GAE/100 g). The trend described by our findings was in agreement with previous literature elucidating the distribution of polyphenols in various parts of *O. ficus-indica* and confirmed that peel is a very interesting source for bioactive compound revalorization. However, not comparable phenolic contents were described among these (by-)products, due to certain variables responsible for consistent variations of such bioactives, such as maturity stage, genetic variability, geopedoclimatic context, and—not least—processing procedure of the *Opuntia* matrix [91,92,93,94].

For the same reasons, literature reported highly variable amounts of single polyphenols among nopal, fruit pulp, peel, and seeds of *O. ficus-indica*, although peculiar compound classes, such as hydroxycinnamic and hydroxybenzoic acids, and flavonoids, constantly characterized these products [24,52,92].

Among phenolic acids, nopal was marked by the predominant *p*-coumaric (498.16 mg/100 g), chlorogenic (179.22 ± 22.49 mg/100 g), and syringic (166.22 mg/100 g) acids. In the fruit pulp, chlorogenic (203.86 mg/100 g), cinnamic (124.33 mg/100 g), and vanillic (111.7 mg/100 g) acids were the most abundant phytoconstituents; while 4-hydroxybenzoic acid (484.95 mg/100 g), cinnamic acid (318.95 mg/100 g), and ferulic acid (127.67 mg/100 g) stood out among the phenolic acids of peels. In line with the low total phenol content, seeds reported very few phenolic acids, including the most abundant ferulic acid (12.53 mg/100 g) (Table 6). In particular, ferulic acid has already emerged as one of the main phenolic compounds, being the precursor of certain feruloyl-sucrose isomers already described in the oil derived from different varieties of *O. ficus-indica* seeds [74].

Differently from the great variability observed for phenolic acids, rutin, and kaempferol were the most abundant compounds in the flavonoid fraction, being significantly different (*p* < 0.05) in nopal (500.05 and 309.38 mg/100 g), fruit pulp (260.45 and 105.83 mg/100 g), peels (818.94 and 270.26 mg/100 g) and seeds (rutin: 20.37 mg/100 g). However, several isorhamnetin and kaempferol glycosides were also found at very high and significantly different levels among the three studied matrices (*p* < 0.05) (Table 6). This is in line with previous studies, highlighting such polyphenols as characteristic bioactives of *O. ficus-indica* [52,95].

## 4. Conclusions

According to the nutritional composition elucidated for the different *Opuntia* (by-)products, it may be concluded that the Tunisian prickly pear cactus could be wisely exploited for encouraging sustainable production, an effective waste management, and for receiving a variety of benefits for human health, in full accordance with the model of the Mediterranean diet.

In line with the literature on *Opuntia*, there is a realistic potential of every by-product from the Tunisian *O. ficus-indica* to be used in the development of functional Mediterranean foods, as well as in the isolation of value-added compounds that may be efficiently employed in food, cosmetic, and pharmaceutical areas. In fact, our findings highlighted that nopal may serve as a precious source of dietary fiber, inorganic elements, such as Mg, K, Mn, and Fe, and monomeric sugars constituting peculiar antihyperlipidemic and antihyperglycemic carbohydrates. The prickly pear peel would be another valuable by-product not only for its minerals and trace essential elements, but also for its polyphenol profile, rich in certain phenolic acids and flavonoids. Finally, the *Opuntia* seeds demonstrated to be marked not only by interesting protein, but also by noticeable lipids and FA composition, which make them already notoriously useful for the production of an edible seed oil with high degree of unsaturation.

As it is a predominant species in the whole Mediterranean area, it is of utmost importance to invest in the research of by-products from *O. ficus-indica* with different geographical origin and/or varieties, with the final aim to valorize its nutritional and functional potential and, not least, move towards circular and profitable economy systems.

## Figures and Tables

**Table 1:** Proximate composition (g/100 g, on a dw basis) of nopal, fruit pulp, peel, and seeds from the Tunisian *O. ficus-indica*. For every product, data are reported in terms of mean ± standard deviation of *n* = 3 samples, where each sample was analyzed three times.

Component	Nopal	Prickly Pear		
		Pulp	Peel	Seeds
Protein	1.36 ± 0.22 ^a^	0.78 ± 0.28 ^b^	1.22 ± 0.49 ^a^	17.34 ± 3.86 ^c^
Lipids	1.15 ± 0.53 ^a^	1.12 ± 0.51 ^a^	5.04 ± 0.57 ^b^	9.65 ± 2.05 ^c^
Ash	18.58 ± 2.19 ^a^	0.28 ± 0.07 ^b^	3.58 ± 0.42 ^c^	1.79 ± 0.26 ^d^
Crude fiber	28.39 ± 3.12 ^a^	4.06 ± 0.92 ^b^	12.54 ± 1.70 ^c^	16.28 ± 2.35 ^c^
Carbohydrates	38.79 ± 3.56 ^a^	74.34 ± 7.38 ^b^	65.23 ± 5.46 ^c^	49.76 ± 6.66 ^d^
Moisture	7.73 ± 1.26 ^a^	16.57 ± 2.98 ^b^	10.12 ± 2.06 ^a^	3.39 ± 0.86 ^c^

^a–d^: different superscript letters in the same row indicate significantly different values for a given parameter (*p* < 0.05 by post hoc Tukey’s HSD test); same superscript letters in the same row indicate not significantly different values for a given parameter (*p* > 0.05 by post hoc Tukey’s HSD test).

**Table 2:** Fatty acid (FA) composition (g/100 g, on a dw basis) of nopal, fruit pulp, peel, and seeds from the Tunisian *O. ficus-indica*. For every product, data are expressed as mean GC-FID peak area ± standard deviation (%, dw) of *n* = 3 samples, where every sample was analyzed three times.

FA	Nopal	Prickly Pear		
		Pulp	Peels	Seeds
C16:0	21.14 ± 1.74 ^a^	16.83 ± 2.33 ^b^	20.76 ± 1.25 ^a^	10.35 ± 1.02 ^c^
C18:0	2.64 ± 0.28 ^a^	4.96 ± 0.45 ^b^	2.65 ± 0.91 ^a^	3.45 ± 0.20 ^c^
C20:0	0.01 ± 0.00 ^a^	0.15 ± 0.07 ^b^	0.01 ± 0.00 ^a^	0.32 ± 0.00 ^b^
*SFA*	*23.79 ± 1.08 ^a^*	*21.94 ± 1.76 ^a^*	*23.42 ± 1.18 ^a^*	*14.12 ± 2.10 ^b^*
C16:1 n-7	0.34 ± 0.12 ^a^	0.97 ± 0.38 ^b^	1.09 ± 0.26 ^b^	1.02 ± 0.23 ^b^
C17:1 n-7	0.57 ± 0.06 ^a^	0.57 ± 0.07 ^a^	0.50 ± 0.29 ^a^	0.12 ± 0.05 ^a^
C18:1 n-9	17.55 ± 2.63 ^a^	23.26 ± 1.31 ^b^	13.56 ± 1.78 ^c^	20.86 ± 1.22 ^b^
C20:1 n-9	0.95 ± 0.28 ^a^	0.25 ± 0.11 ^a^	1.05 ± 0.09 ^b^	0.42 ± 0.26 ^a^
*MUFA*	*19.41 ± 2.98 ^a^*	*25.05 ± 1.66 ^b^*	*16.21 ± 1.85 ^a^*	*22.42 ± 0.95 ^c^*
C18:2 n-6	36.44 ± 3.81 ^a^	48.97 ± 4.86 ^b^	47.85 ± 3.86 ^b^	61.11 ± 5.55 ^c^
C18:3 n-3	19.64 ± 1.08 ^a^	3.26 ± 0.76 ^b^	11.44 ± 0.19 ^c^	0.29 ± 0.09 ^d^
C20:4 n-6	0.61 ± 0.19 ^a^	0.75 ± 0.09 ^a^	0.92 ± 0.14 ^a^	0.32 ± 0.08 ^b^
*PUFA*	*56.69 ± 5.89 ^a^*	*52.93 ± 6.09 ^a^*	*60.21 ± 4.67 ^a^*	*61.71 ± 3.05 ^a^*
*H/H*	*3.48*	*5.45*	*3.50*	*7.95*
*AI*	*1.11*	*0.86*	*1.09*	*0.49*
*TI*	*0.27*	*0.43*	*0.35*	*0.32*
*ω-6/ω-3*	*1.89*	*15.26*	*4.27*	*211.83*

^a–d^: different superscript letters in the same row indicate significantly different values for a given parameter (*p* < 0.05 by post hoc Tukey’s HSD test); same superscript letters in the same row indicate not significantly different values for a given parameter (*p* > 0.05 by post hoc Tukey’s HSD test).

**Table 3:** Minerals (mg/100 g, dw), essential (mg/Kg product, dw), and potentially toxic (µg/Kg product, dw) trace elements of nopal, fruit pulp, peel, and seeds from the Tunisian *O. ficus-indica*. For every product, data are expressed as mean ± standard deviation of *n* = 3 samples, where every sample was analyzed three times. Limit of detection (LOD) of As = 0.015 µg/Kg, Cd = 0.008 µg/Kg and Pb = 0.012 µg/Kg.

Element	Nopal	Prickly Pear		
		Pulp	Peel	Seed
	Minerals (mg/100 g, dw)			
Na	144.54 ± 21.09 ^a^	2.58 ± 0.26 ^b^	114.29 ± 15.95 ^c^	14.09 ± 0.17 ^d^
Mg	493.57 ± 87.73 ^a^	152.84 ± 29.38 ^b^	345.19 ± 55.67 ^c^	427.35 ± 76.17 ^a^
K	6949.57 ± 1039.89 ^a^	187.05 ± 15.85 ^b^	1820.83 ± 20.33 ^c^	214.36 ± 18.93 ^b^
	Essential trace elements (mg/Kg, dw)			
Mn	59.73 ± 12.96 ^a^	0.78 ± 0.07 ^b^	46.86 ± 6.13 ^a^	1.56 ± 0.09 ^c^
Fe	23.15 ± 3.07 ^a^	2.36 ± 0.16 ^b^	15.27 ± 1.24 ^c^	4.99 ± 0.35 ^d^
Cu	0.05 ± 0.01 ^a^	0.21 ± 0.03 ^b^	11.55 ± 1.58 ^c^	0.46 ± 0.12 ^b^
Zn	11.16 ± 1.25 ^a^	5.09 ± 0.28 ^b^	24.96 ± 2.20 ^c^	31.58 ± 3.60 ^d^
	Potentially toxic trace elements (µg/Kg, dw)			
Cr	41.50 ± 5.13 ^a^	20.14 ± 1.44 ^b^	15.41 ± 2.62 ^c^	11.92 ± 1.03 ^c^
Ni	47.14 ± 5.59 ^a^	55.46 ± 3.10 ^a^	34.79 ± 1.99 ^b^	33.28 ± 1.41 ^b^
As	<LOD	<LOD	<LOD	24.15 ± 1.40
Cd	25.62 ± 2.13	<LOD	<LOD	<LOD
Pb	22.37 ± 1.81 ^a^	23.72 ± 2.04 ^a^	13.77 ± 3.02 ^b^	11.28 ± 2.76 ^b^

^a–d^: different superscript letters in the same row indicate significantly different values for a given parameter (*p* < 0.05 by post hoc Tukey’s HSD test); same superscript letters in the same row indicate not significantly different values for a given parameter (*p* > 0.05 by post hoc Tukey’s HSD test).

**Table 4:** Sugars (g/100 g, dw) of nopal, fruit pulp, peel, and seeds from the Tunisian *O. ficus-indica*. For every product, data are expressed as mean ± standard deviation of *n* = 3 samples, where every sample was analyzed three times.

Sugar	Cladode	Prickly Pear		
		Pulp	Peel	Seed
Glucose	8.75 ± 2.16 ^a^	42.57 ± 13.88 ^b^	21.79 ± 4.94 ^c^	19.37 ± 4.25 ^d^
Galactose	9.76 ± 1.13 ^a^	2.34 ± 0.51 ^b^	15.67 ± 3.21 ^c^	0.56 ± 0.03 ^d^
Xylose	7.78 ± 0.91 ^a^	6.78 ± 1.79 ^a^	5.48 ± 0.72 ^a^	22.29 ± 3.87 ^b^
Arabinose	13.78 ± 3.81 ^a^	13.56 ± 3.31 ^a^	4.22 ± 1.05 ^b^	2.27 ± 0.29 ^c^
Mannose	1.92 ± 0.45 ^a^	4.75 ± 1.73 ^b^	6.96 ± 1.78 ^b^	1.34 ± 0.38 ^a^

^a–d^: different superscript letters in the same row indicate significantly different values for a given parameter (*p* < 0.05 by post hoc Tukey’s HSD test); same superscript letters in the same row indicate not significantly different values for a given parameter (*p* > 0.05 by post hoc Tukey’s HSD test).

**Table 5:** Total polyphenols (mg GAE/100 g, dw) of different (by-)products from the Tunisian *O. ficus-indica*. For every product, data are expressed as mean ± standard deviation of *n* = 3 samples, where every sample was analyzed three times.

Total Polyphenols	Cladode	Prickly Pear		
		Pulp	Peel	Seeds
	4235.27 ± 68.15 ^a^	2581.68 ± 45.21 ^b^	4785.36 ± 73.16 ^c^	108.36 ± 10.15 ^d^

^a–d^: different superscript letters in the same row indicate significantly different values for a given parameter (*p* < 0.05 by post hoc Tukey’s HSD test); same superscript letters in the same row indicate not significantly different values for a given parameter (*p* > 0.05 by post hoc Tukey’s HSD test).

**Table 6:** Single polyphenols (mg/100 g, dw) of various parts from the Tunisian *O. ficus-indica*. For every product, data are expressed as mean ± standard deviation of *n* = 3 samples, where every sample was analyzed three times.

Polyphenol	λ_max_ (nm)	[M − H] (*m/z*)	Cladode	Prickly Pear		
				Pulp	Peel	Seeds
Gallic acid	214,270	169	33.27 ± 3.55 ^a^	11.68 ± 1.40 ^b^	65.50 ± 2.76 ^c^	<LOD
Protocatechuic acid	218,260,295	153	50.61 ± 4.47 ^a^	21.89 ± 2.21 ^b^	9.61 ± 1.19 ^c^	<LOD
4-hy droxybenzoic acid	255	137	9.44 ± 1.26 ^a^	16.77 ± 1.56 ^b^	484.95 ± 8.73 ^c^	1.37 ± 0.11 ^d^
Vanillic acid	220,260,295	167	84.15 ± 10.14 ^a^	111.77 ± 17.13 ^a^	24.86 ± 3.57 ^b^	<LOD
Syringic acid	220,275	197	166.22 ± 18.99 ^a^	3.23 ± 0.62 ^b^	59.49 ± 4.09 ^c^	3.78 ± 0.75 ^b^
Cinnamic acid	321	147	42.42 ± 3.10 ^a^	124.33 ± 8.78 ^b^	318.95 ± 11.99 ^c^	<LOD
Chlorogenic acid	324	353	179.22 ± 22.49 ^a^	203.86 ± 23.36 ^a^	28.84 ± 4.33 ^b^	<LOD
Caffeic acid	210,272,328	179	23.50 ± 5.09 ^a^	10.64 ± 2.38 ^b^	98.88 ± 2.97 ^c^	<LOD
*p*-coumaric acid	225,310	163	498.16 ± 58.38 ^a^	4.00 ± 0.93 ^b^	57.79 ± 2.22 ^c^	<LOD
Ferulic acid	230,320	193	69.92 ± 9.23 ^a^	251.16 ± 7.46 ^b^	127.67 ± 5.82 ^c^	12.53 ± 3.22 ^d^
Sinapic acid	235,322	223	20.21 ± 2.04 ^a^	85.71 ± 5.27 ^b^	46.17 ± 3.39 ^c^	<LOD
*Total phenolic acids*	*-*	*-*	*1177.12 ± 41.89 ^a^*	*845.01 ± 46.78 ^b^*	*1322.68 ± 27.22 ^c^*	*17.68 ± 5.22 ^d^*
Rutin	257,354	609	500.05 ± 33.39 ^a^	260.45 ± 10.15 ^b^	818.94 ± 44.86 ^c^	20.37 ± 5.87 ^d^
Isorhamnetin 3-*O*-glucoside	250,342	477	149.71 ± 10.13 ^a^	184.14 ± 14.91 ^b^	223.66 ± 14.44 ^c^	<LOD
Kaempferol-3-*O*-rutinoside	266,300,346	593	253.46 ± 48.05 ^a^	171.52 ± 12.78 ^b^	159.22 ± 27.65 ^b^	<LOD
Kaempferol-3-*O*-glucoside	262,362	447	479.77 ± 31.90 ^a^	132.11 ± 9.62 ^a^	512.44 ± 42.58 ^c^	<LOD
Isorhamnetin-3-*O*-rutinoside	250,270,342	623	703.33 ± 28.45 ^a^	271.39 ± 25.59 ^b^	254.51 ± 31.03 ^b^	<LOD
Quercetin	205,254,370	301	48.77 ± 7.70 ^a^	63.60 ± 8.24 ^b^	605.28 ± 22.71 ^c^	32.33 ± 11.09 ^d^
Luteolin	365	285	49.29 ± 2.83 ^a^	71.72 ± 5.27 ^b^	80.12 ± 6.71 ^b^	<LOD
Apigenin	268,334	269	167.06 ± 14.54 ^a^	59.95 ± 6.51 ^b^	60.89 ± 2.02 ^b^	<LOD
Kaempferol	266,368	285	309.38 ± 50.83 ^a^	105.83 ± 10.62 ^b^	270.26 ± 14.19 ^c^	<LOD
*Total flavonoids*	*-*	*-*	*2660.84 ± 68.48 ^a^*	*1320.68 ± 38.30 ^b^*	*2985.30 ± 30.10 ^c^*	*52.70 ± 7.36 ^d^*
*Total polyphenols*	*-*	*-*	*3837.96 ± 110.37 ^a^*	*2165.69 ± 8.41 ^b^*	*4307.98 ± 17.12 ^c^*	*70.38 ± 14.37 ^d^*

^a–d^: different superscript letters in the same row indicate significantly different values for a given parameter (*p* < 0.05 by post hoc Tukey’s HSD test); same superscript letters in the same row indicate not significantly different values for a given parameter (*p* > 0.05 by post hoc Tukey’s HSD test).

## Data Availability

Not applicable.

## Supplementary Materials

The following supporting information can be downloaded at: https://www.mdpi.com/article/10.3390/foods11020155/s1, Paragraph: Analytical validation of ICP-MS and HPLC-DAD-MS method; Table S1: Performance of the ICP-MS method in terms of linearity, LOD, LOQ, and intra- and interday repeatability (*n* = 6), and accuracy tested on representative samples of nopal, fruit pulp and peel (*n* = 5). LOD = limit of detection; LOQ = Limit of Quantification; RSD = relative standard deviation. Table S2. Performance of the LC-MS method in terms of linearity, LOD, LOQ, and intra- and interday repeatability (*n* = 6), and accuracy tested on representative samples of nopal, fruit pulp and peel (*n* = 5). Analytical performance of the LC-MS method. LOD = limit of detection; LOQ = Limit of Quantification; RSD = relative standard deviation.
