# Supplementary material for: Chemical Characterization of Different Products from the Tunisian Opuntia ficus-indica (L.) Mill
Source: Foods. 2022 Jan 7;11(2):155. doi: 10.3390/foods11020155 (PMC8775233; doi:10.3390/foods11020155)
Supplement: Supplementary file 1 [file foods-11-00155-s001.zip › foods-1503739-supplementary.pdf]

Supplementary Materials

Analytical validation of ICP-MS and HPLC-DAD-MS method

Methods of analysis were validated by reference standards in terms of linearity, limit of detection (LOD) and quantification (LOQ), accuracy, intra- and interassay variability. Linearity was assessed by means of calibration curves of each analyte, constructed according to the linear least-square regression method, and the obtained correlation coefficients were comprised between 0.9928 and 0.9998 for inorganic elements (table S1), and ranged from 0.9947 to 0.9998 for polyphenols (table S2). LOD and LOQ were determined as 3 and 10 times, respectively, the blank standard deviation (n=6) divided by the slope of the analyte calibration curve. For inorganic elements, LODs ranged from 0.008 ppb to 0.18 ppb and, accordingly, LOQs were between 0.02 ppb and 0.63 ppb, respectively for Cd and Zn (table S1). For polyphenols, the lowest LOD and LOQ were obtained for isorhamnetin 3-*O*-glucoside (0.087 ppm and 0.275 ppm); while ferulic acid was characterized by the highest values (LOD = 0.48 ppm and LOQ = 1.55 ppm) (table S2).

Due to the unavailability of (certified) reference materials, the accuracy was estimated by the surrogate recovery. In a separate experiment, representative samples of nopal, fruit pulp, peel and seeds were spiked with known amounts of each investigated analyte (i.e. inorganic element or polyphenol) and analyzed in five replicates alongside the same unspiked sample. The mean difference between these two results, corresponding to the recovered part of the spiked analyte, was compared with the known spiked amount. Acceptable recoveries were obtained for polyphenols (from 94.3% [Mn in seeds] to 104.2% [Ni in nopal]) and for polyphenols (from 85.4% [4-hydroxybenzoic acid in seeds] to 110.4%

[Rutin in nopal]) (tables S1 and S2). Finally, precision was determined as the relative standard deviation (RSD%) of the recovery of every analyte, calculated in the same day (intraday precision) and in five consecutive days (interday precision). For inorganic elements, intraday precision was assessed between 1.93% (Na) and 5.77% (Cr), while interday precision was below 7.99% (Cu) (table S1). For polyphenols, intraday precision varied from 0.5% (caffeic acid) to 5.3% (isorhamnetin 3-*O*-glucoside); while interday precision was from 1.8% (caffeic acid) to 7.6% (kaempferol 3-O-glucoside) (table S2).

Table S1. Performance of the ICP-MS method in terms of linearity, LOD, LOQ, and intra- and interday repeatability (*n*=6), and accuracy tested on representative samples of nopal, fruit pulp and peel (*n*=5). LOD = limit of detection; LOQ = Limit of Quantification; RSD = relative standard deviation.

Element	a	b	R^2	LOD (ng g^{-1})	LOQ (ng g^{-1})	Precision (RSD%)		Accuracy (%)			
	intercept	slope				Intraday	Interday	Nopal	Fruit pulp	Peel	Seeds
^{23}Na	$1.39 \cdot 10^8$	$1.41 \cdot 10^8$	0.9999	0.17	0.61	1.93	2.72	99.7	98.9	102.3	97.5
^{24}Mg	$3.31 \cdot 10^8$	$5.74 \cdot 10^6$	0.9992	0.13	0.49	3.44	4.06	102.4	98.5	99.5	105.2
^{39}K	$4.59 \cdot 10^7$	$6.53 \cdot 10^8$	0.9999	0.15	0.52	2.81	3.44	101.4	100.3	101.7	103.2
^{52}Cr	$1.04 \cdot 10^4$	$2.46 \cdot 10^3$	0.9999	0.018	0.06	5.77	6.52	95.7	95.1	98.9	100.9
^{55}Mn	$9.28 \cdot 10^4$	$1.20 \cdot 10^4$	0.9998	0.16	0.58	3.95	4.43	96.1	96.4	102.3	94.3
^{56}Fe	$2.99 \cdot 10^4$	$3.80 \cdot 10^6$	0.9995	0.15	0.54	3.03	5.12	99.7	98.9	100.7	97.6
^{60}Ni	$3.34 \cdot 10^3$	$1.89 \cdot 10^3$	0.9996	0.009	0.03	4.51	5.20	104.2	101.8	100.7	104.1
^{63}Cu	$8.55 \cdot 10^3$	$2.86 \cdot 10^4$	0.9991	0.14	0.49	4.82	7.99	99.8	99.1	95.5	92.9
^{66}Zn	$2.94 \cdot 10^3$	$3.26 \cdot 10^4$	0.9993	0.18	0.63	4.92	8.15	95.9	96.7	94.5	97.5
^{75}As	$1.91 \cdot 10^3$	$2.91 \cdot 10^2$	0.9992	0.015	0.05	4.10	5.87	100.5	98.9	100.3	98.1
^{111}Cd	$8.93 \cdot 10^3$	$2.39 \cdot 10^2$	0.9998	0.008	0.02	3.12	3.52	95.4	97.1	98.0	101.4
^{208}Pb	$6.41 \cdot 10^4$	$8.36 \cdot 10^3$	0.9997	0.012	0.04	4.07	5.91	96.9	100.5	94.4	97.8

Table S2. Performance of the LC-MS method in terms of linearity, LOD, LOQ, and intra- and interday repeatability (*n*=6), and accuracy tested on representative samples of nopal, fruit pulp and peel (*n*=5). Analytical performance of the LC-MS method. LOD = limit of detection; LOQ = Limit of Quantification; RSD = relative standard deviation.

Analyte	Regression model	R^2	LOD (ppm)	LOQ (ppm)	Precision (RSD %)		Accuracy(%)			
					Intraday	Interday	Nopal	Fruit pulp	Peel	Seeds
Hydroxybenzoic acids										
**Gallic acid**	y = 8311.14x - 1234.10	0.9983	0.13	0.38	4.2	5.0	94.6	96.7	98.9	93.4
**Protocatechuic acid**	y = 9035.30x - 1164.03	0.9979	0.23	0.73	3.2	5.9	89.1	92.3	98.7	90.2
**4-Hydroxybenzoic acid**	y = 7534.05x - 1683.43	0.9992	0.096	0.32	2.9	6.3	87.9	89.2	95.3	85.0
**Vanillic acid**	y = 8449.24x - 1223.72	0.9994	0.17	0.50	1.5	2.8	95.7	96.9	104.7	89.7
**Syringic acid**	y = 6938.23x - 634.28	0.9971	0.32	0.95	1.8	2.1	93.9	97.4	102.5	97.4
Hydroxycinnamic acids										
**Cinnamic acid**	y = 2859.38x – 729.56	0.9985	0.32	1.02	2.5	5.7	97.6	95.3	89.8	90.2
**Chlorogenic acid**	y = 7652.48x-1651.29	0.9996	0.28	0.92	0.8	3.5	98.7	105.34	97.5	98.6
**Caffeic acid**	y = 13117.21x-1531.41	0.9973	0.15	0.48	0.5	1.8	107.5	96.8	96.3	93.0
**Ferulic acid**	y = 1532.33x-248.13	0.9986	0.48	1.55	3.9	5.2	89.5	91.8	92.1	105.3
**Sinapic acid**	y = 48231.52x – 18207.25	0.9959	0.25	0.82	3.8	6.4	99.6	101.9	98.8	88.5
***p*-coumaric acid**	y = 65402.37x – 25178.82	0.9990	0.10	0.34	2.7	4.8	92.6	89.8	99.2	85.4
Flavonoids										
**Rutin**	y = 10183.55x – 3808.49	0.9985	0.31	1.03	1.3	3.5	110.4	102.5	103.6	102.7
**Isorhamnetin 3-*O*-glucoside**	y = 21913.22x – 4421.33	0.9991	0.087	0.275	5.3	7.4	95.8	92.5	104.4	90.6
**Kaempferol-3-*O*-rutinoside**	y = 59036.39x – 8787.23	0.9986	0.23	0.68	4.4	6.1	91.7	94.8	93.6	91.9
**Kaempferol 3-*O*-glucoside**	y = 6532.77x – 3433.71	0.9947	0.31	0.99	3.7	7.6	90.8	93.6	97.1	90.2
**Isorhamnetin-3-*O*-rutinoside**	y = 104319.51x – 43219.57	0.9995	0.45	1.49	3.7	5.5	91.2	96.5	95.2	94.2

**Quercetin**	y = 9810.23x - 763.13	0.9991	0.38	1.25	2.1	4.0	98.6	94.1	102.3	107.3
**Luteolin**	y = 8451.84x - 2231.45	0.9998	0.098	0.32	2.3	3.0	94.6	89.2	102.3	95.7
**Apigenin**	y = 9150.32x - 1464.36	0.9970	0.25	0.84	1.8	2.9	87.5	96.3	92.9	97.7
**Kaempferol**	y = 10521.74x - 1256.34	0.9992	0.19	0.61	1.1	2.7	95.9	99.7	103.1	96.2
